# Influence of Starvation on Biochemical, Physiological, Morphological, and Transcriptional Responses Associated with Glucose and Lipid Metabolism in the Liver of Javelin Goby (*Synechogobius hasta*)

**DOI:** 10.3390/ani14182734

**Published:** 2024-09-21

**Authors:** Xiangyu Cui, Xiaoyang Huang, Xiangning Chen, Honghui Li, Yanru Wu, Zikui Yang, Zhiyu Liu, Rui Feng, Jianhe Xu, Chaoqing Wei, Zhujin Ding, Hanliang Cheng

**Affiliations:** 1 Jiangsu Key Laboratory of Marine Bioresources and Environment, Jiangsu Key Laboratory of Marine Biotechnology, Jiangsu Ocean University, Lianyungang 222005, China2023210123@jou.edu.cn (X.H.);; 2 Key Laboratory of Cultivation and High-Value Utilization of Marine Organisms, Fisheries Research Institute of Fujian, Xiamen 361000, China; 3 Co-Innovation Center of Jiangsu Marine Bio-Industry Technology, Jiangsu Ocean University, Lianyungang 222005, China; 4 College of Marine Science and Fisheries, Jiangsu Ocean University, Lianyungang 222005, China; 5 Wuxi Fisheries College, Nanjing Agricultural University, Wuxi 214081, China

**Keywords:** *Synechogobius hasta*, food deprivation, liver, glucose metabolism, lipid metabolism

## Abstract

**Simple Summary:**

When faced with starvation, fish can consume energy reserves and adaptively modify the metabolic responses of main energy substrates for fundamental activity. Carbohydrates and lipids are mobilized preferentially in most fish under starvation. However, the mobilization of endogenous energy in starving fish varies with the species, tissue, and fasting duration. Liver tissue is the primary tissue for energy reserves in multiple fish, particularly lipid-associated components. Therefore, this study was conducted to investigate the phenotypic changes in physiological, metabolic, histological, and molecular characteristics of glucose and lipid metabolism in the liver of *Synechogobius hasta* experiencing different starvation periods. Hepatic glycogen and triglycerides were concurrently mobilized in *S. hasta* during starvation, which was accompanied by consistent changes in liver intermediary metabolism enzyme activities and an increase in liver antioxidant capacity to some extent. *S. hasta* livers exhibited evident vacuolations, larger intercellular spaces, and other microstructural abnormalities from the third day of starvation. Additionally, fasting not only transcriptionally attenuated glucose and lipid anabolism, but also enhanced the catabolic pathway and fatty acid transport in *S. hasta* liver. These findings may provide preliminary data for further assessing the energy strategy to cope with starvation and the underlying mechanism in *S. hasta* and other fish species.

**Abstract:**

In this study, the influence of fasting on hepatic glucose and lipid metabolism was explored by examining biochemical, antioxidative, and morphological indicators and transcriptional expression in the liver of javelin goby (*Synechogobius hasta*) after 0, 3, 7, or 14 days of starvation. Marked reductions in hepatic glycogen and triglycerides occurred from the seventh day of starvation until the end of the trial (*p* < 0.05). However, no alterations in hepatic cholesterol or protein were detected throughout the entire experiment (*p* > 0.05). During fasting, the activities of pyruvate kinase, lactate dehydrogenase, and glycogen phosphorylase a all rose firstly and then fell (*p* < 0.05). The activities of hepatic fatty acid synthase and acetyl-CoA carboxylase were minimized to their lowest levels at the end of food deprivation (*p* < 0.05), while lipase was elevated after 7–14 days of fasting (*p* < 0.05). Catalase, glutathione, and the total antioxidative capacity were increased and maintained their higher values in the later stage of fasting (*p* < 0.05), whereas malondialdehyde was not significantly changed (*p* > 0.05). Hepatic vein congestion, remarkable cytoplasmic vacuoles, and irregular cell shape were present in *S. hasta* which endured 3–7 days of fasting and were less pronounced when food shortage was prolonged. In terms of genes associated with glucose and lipid metabolism, the hepatic phosphofructokinase gene was constantly up-regulated during fasting (*p* < 0.05). However, the mRNA levels of glycogen synthase and glucose-6-phosphatase were obviously lower when the food scarcity extended to 7 days or more (*p* < 0.05). Fatty acid synthase, stearoyl-CoA desaturase 1, and peroxisome proliferator-activated receptor γ were substantially down-regulated in *S. hasta* livers after 7–14 days of food deprivation (*p* < 0.05). However, genes involved in lipolysis and fatty acid transport were transcriptionally enhanced to varying extents and peaked at the end of fasting (*p* < 0.05). Overall, starvation lasting 7 days or more could concurrently mobilize hepatic carbohydrates and fat as energy resources and diminished their hepatic accumulation by suppressing biosynthesis and enhancing catabolism and transport, ultimately metabolically and structurally perturbing the liver in *S. hasta*. This work presents preliminary data on the dynamic characteristics of hepatic glucose and lipid metabolism in *S. hasta* in response to starvation, which may shed light on the sophisticated mechanisms of energetic homeostasis in fish facing nutrient unavailability and may benefit the utilization/conservation of *S. hasta*.

## 1. Introduction

Starvation stress triggered by various nutrient or environmental factors is a critical challenge for all organisms, including fish [1,2,3]. In cases of food scarcity, fish can consume and allocate energy reserves stored in tissues for fundamental activity, paralleling adaptive adjustments to energy metabolism [1,2,3]. The liver is a primary site for energy reserves in multiple fish, particularly lipid-associated components, like triglycerides [1,4,5]. It was further verified to dominate the physiological and metabolic adjustments of energy homeostasis in fish facing starvation [1]. Certainly, the energy metabolism of muscular, gastrointestinal tissue, and other visceral tissues in aquatic animals, is also markedly influenced by a lack of available nutrients [1,4,5,6,7]. Nevertheless, the energy stores available in the extrahepatic organs are limited and cannot adequately cover the energy demand of a whole fish during longer periods of sustained starvation [1,8], eventually causing energy dysmetabolism. Carnivorous fish have limited glucose utilization ability compared with herbivorous fish [9], and aquafeed with excessively high carbohydrate and high fat levels has been reported to cause poor growth, low feed utilization, fatty liver symptoms, and other negative effect related to liver health in various fish species [10,11,12]. Therefore, investigating the physiological processes of energy metabolism modulation in liver is a prerequisite to understanding energy strategies in fish during starvation and providing a theoretical fundament for a healthier, lower-cost, and greater performance-improved aquaculture. However, the metabolic rules of energy reserves during food shortages, particularly those related to intrahepatic energy substrates and the intrinsic mechanism, are less well defined in fish than in mammals.

Most fish prefer to use body reserves of lipids and carbohydrates as energy supplies when they have insufficient access to food [1,2,5,13], particularly during short-term fasting. Protein catabolism mainly occurs when endogenous glucose and fat are completely exhausted during prolonged starvation [1,5,13]. Only a few fish species preferentially expend protein in the initial stage of food deprivation [1,5,14]. Furthermore, lipids and related substances are known to have a higher energy density than carbohydrates and protein [15], and they seem to play a more prominent role in maintaining energy homeostasis in many fish that are not feeding [1,2,4,16]. However, the mobilization of endogenous energetic materials and its dynamic features in starving fish differ according to fish species, fasting duration, and storage sites [1,5].

The javelin goby (*Synechogobius hasta*) is a well-known marine fish with the favorable economic prospects and great ecological significance on the Pacific Coast, including the sea region of Lianyungang, Jiangsu, China [17,18,19]. It possesses rapid growth, desirable taste, high disease resistance, and wide adaptability to various salinities [8,20]. Additionally, *S. hasta* has been employed experimentally as a model of lipid homeostasis in fish because of its substantial hepatic fat deposits in natural seawater conditions [20]. In our previous studies, earlier mobilization of muscle glycogen compared to muscle fat occurred in starved *S. hasta*, accompanied by transcriptional down-regulation of muscular lipid metabolism [17]. However, brief fasting stimulated the up-regulation of fat breakdown and fatty acid transport in the intestine of *S. hasta* [8]. Given the inter-species and inter-tissue variability in the utilization performance of the three main physiological fuels in starving animals [1,2,5], as well as the core function of the liver in the metabolic homeostasis of fish, whether the preferential mobilization of carbohydrates would occur in the liver of *S. hasta* facing starvation in the same way as reported in other food-deprived fish [1,5,9,13] is not clear. Furthermore, the metabolic adjustments of glucose and lipid storage in the liver of *S. hasta* under fasting and their molecular mechanisms need to be further elucidated. Hence, this work aimed to evaluate adaptive alterations in the physiological, metabolic, and molecular responses of hepatic glucolipid metabolism, as well as hepatic morphological changes, in *S. hasta* suffering 0, 3, 7, or 14-day food deprivation. Our results may clarify the energy strategies and the underlying mechanism by which fish cope with food shortages and may provide reference data for further utilization of *S. hasta* and other marine fish resources.

## 2. Materials and Methods

### 2.1. Ethics Statement

All experimental operations involving *S. hasta* were approved by the Animal Care and Use Committee of Jiangsu Ocean University (Approval No. 2020-37) and obeyed the Ethical Guidelines for the Care and Use of Laboratory Animals in China.

### 2.2. Fish Maintenance and Experimental Treatment

Javelin goby were obtained from a local sea area in Lianyungang, Jiangsu province, China, and were quickly shifted to a fish holding tank (240 L) at the Jiangsu Key Laboratory of Marine Biotechnology, Jiangsu Ocean University (Lianyungang, Jiangsu, China). Before the commencement of the fasting trial, all fish were kept in the 240 L tank and acclimated to the ambient laboratory conditions for two weeks according to previous studies [8,17,18,19]. Briefly, the fish were fed minced shrimp scraps each day (feeding amount: 5–6% of fish weight; feeding times: 8:30 and 17:00). Shrimp residue and feces were cleaned promptly, and half of the water was replaced daily. The aquatic environment was monitored regularly during the entire experiment, and the main quality parameters of the artificial seawater were maintained as follows: temperature, 19.5–20.5 °C; salinity, 18.5–21.5‰; dissolved oxygen, ≥6 mg/L; pH, 7.8–8.3; NH4-N, ≤0.075 mg/L; photoperiod, 12 h of light and 12 h of dark.

At the start of the starvation experiment, sixty healthy *S. hasta* with similar specifications (body weight: 125.14 ± 10.53 g; body length: 33.67 ± 3.08 cm) were randomly partitioned into four groups (five fish/group, three tanks/group, and 240 L/tank), i.e., one control group and three starvation groups. The fish in the control group continued to be supplied with minced shrimp scraps twice daily throughout the experimental period, while the *S. hasta* in the three starvation groups were food deprived for 3 (feeding from day 1 to day 11, fasting from day 12 to day 14), 7 (feeding from day 1 to day 7, fasting from day 8 to day 14), or 14 days (fasting from day 1 to day 14). The three starvation groups could be referred to as S3 (starved for 3 days), S7 (starved for 7 days), and S14 (starved for 14 days), respectively. This fasting trial lasted 14 days, during which the aquatic environment was kept uniform according to the aforementioned acclimation conditions.

### 2.3. Sampling

At the termination of the 14-day fasting experiment, the *S. hasta* were treated with 100 mg/L MS-222 (Beijing Green Hengxing Biological Technology Co., Ltd., Beijing, China) and mechanically dissected on ice to obtain liver tissue. Three liver specimens from each group were immediately soaked in 10% neutral formaldehyde for later histological processing. The rest of the *S. hasta* liver samples were stripped of impurities, washed thoroughly with ice-cold sterilized PBS, individually weighed, rapidly placed in liquid nitrogen, and stored at −80 °C for follow-up analyses.

### 2.4. Hepatic Biochemical Parameters, Intermediate Metabolic Enzymes, and Antioxidant Indices

Each liver sample was homogenized with sterilized PBS (tissue weight/PBS volume =1:9 mg/μL) in an ice bath using a high-throughput grinding instrument (TGrinder H24R, Tiangen Biotech Co., Ltd., Beijing, China), followed by centrifugation at 4 °C and 2500 rpm/min for 10 min. The extracted supernatant was further evaluated using a biochemical assay.

Hepatic biochemical parameters, including glycogen (GLY), total triglycerides (TG), total cholesterol (TC), and total protein (TP), were determined using commercial kits (Nanjing Jiancheng Bioengineering Institute, Nanjing, China) according to the manufacturer’s standard protocol.

Regarding intermediate metabolic enzyme activity, pyruvate kinase (PK) and lactate dehydrogenase (LDH) were examined using commercial enzymatic detection kits (Nanjing Jiancheng, Nanjing, China) as per the corresponding protocols. Glycogen phosphorylase a (GPa) activity was tested with a GPa activity assay kit (BC3345, Solarbio Science & Technology Co., Ltd., Beijing, China). Fatty acid synthase (FAS) activity was determined manually, as previously described [21]. Lipase (LPS) was assessed based on 1, 2-O-dilauryl-rac-glycero-3-glutaric acid-(6-methyl-resorufin) ester as a substrate according to previous research [22]. To quantify hepatic acetyl-CoA carboxylase (ACC) activity, a fish ACC ELISA kit from Nanjing Jiancheng (Nanjing, China) was employed.

The activities of antioxidative enzymes, including superoxide dismutase (SOD), catalase (CAT), and glutathione peroxidase (GSH-Px); and the levels of total antioxidative capacity (T-AOC), glutathione (GSH), and malondialdehyde (MDA) in the *S. hasta* liver were quantified with corresponding kits (Jiancheng, Nanjing, China) according to the recommended assay procedures.

All detection procedures were conducted in a 96-well microplate on a Multiskan FC photometer (Thermo Scientific, Waltham, MA, USA) at the respective wavelengths.

### 2.5. Hepatic Histochemical Staining and Histomorphological Observation

The preparation of hepatic paraffin sections and histochemical staining were conducted according to previous research with some alterations [10]. Freshly dissected liver tissues were washed with ice-cold sterilized PBS, trimmed into appropriate shapes, and treated with 10% neutral buffered formalin overnight for immersion fixation. The fixed liver tissues were dehydrated in the gradient alcohol (75–100%), cleared in a xylene solution, and placed in melted paraffin for embedding. After solidification, the embedded samples (1.5 × 1.5 × 0.3 cm) were sectioned at 4–6 μm using a rotary microtome (RM2235, Leica Biosystems Nussloch GmbH, Heidelberger, Germany), placed on microscope slides, and baked in a thermostatted oven at 50–60 °C.

Prior to staining, the paraffin liver sections were dewaxed using xylene, then rehydrated in gradient alcohol and water. For hematoxylin and eosin (HE) staining, the sectioned liver samples were treated with an HE solution at room temperature for 5 min. After dehydration in gradient alcohol, xylene was used to make the slices transparent. They were then mounted with neutral gum for histological observation. All stained sections were viewed using a Nikon 90i Microscope (Nikon, Tokyo, Japan).

### 2.6. Quantitative Real-Time Polymerase Chain Reaction (qRT-PCR) Analysis

The relative expression of target genes was evaluated using qRT-PCR according to our previous protocol with modifications [8,12]. Total RNA was isolated from the *S. hasta* livers using RNAiso Plus (Takara Biotechnology Co., Ltd., Dalian, China) and was then evaluated qualitatively and quantitatively using 1% agarose gel electrophoresis and a spectrophotometer (NanoDrop2000, Thermo Fisher Scientific, Wilmington, DE, USA), respectively. Hepatic RNA (1 μg) was reverse transcribed to obtain cDNA using a PrimeScript^®^ RT Reagent Kit with gDNA Eraser (TaKaRa, Dalian, China) according to the manufacturer’s protocol. A qRT-PCR assay was performed on a LightCycler^®^ 96 system (Roche Applied Science, Mannheim, Germany) using a TB Green^®^ Premix Ex Taq™ II kit (RR820B, Takara, Dalian, China). The 20 μL qRT-PCR reaction system contained 1 µL of cDNA, 10 μL of TB Green Premix Ex Taq II (2×), 1 µL of the forward primer (10 µmol/L), 1 µL of the reverse primer (10 µmol/L), and 7 µL of RNase-free ddH_2_O. The amplification program was run as follows: initial denaturation at 95 °C for 30 s; 40 cycles of 95 °C for 5 s and 60 °C (or 57 °C) for 30 s; and a final sequence of 95 °C for 15 s, 60 °C for 60 s, and 95 °C for 15 s. All qRT-PCR reactions were performed in triplicate for each sample, and a reaction without a cDNA fragment was used as a negative control. *β-actin* was employed as an internal control gene in this study according to the dissociation curves and amplification efficiencies of three candidate references (*18s*, *α-tubulin*, and *β-actin*). The relative expression level of a target gene was calculated as its amount relative to the internal reference gene according to the 2-ΔΔCt method. Specific primer pairs were synthesized by Sangon (Shanghai, China), and detailed information related to these primers is presented in Table 1.

### 2.7. Statistical Methods

All the data were pooled and are expressed as means ± standard deviations (SD) with *n* = 4–5 for hepatic antioxidant parameters, while data are presented as mean ± SD with *n* = 5 for the detection of other indicators. Prior to the analysis of differences in mean values, the normal distribution of quantitative variables was determined using the Shapiro–Wilk test. Normally distributed data were further tested for homogeneity of variance using the Levene test, while non-normally distributed data were tested for equal variance using the Brown–Forsythe test. A one-way analysis of variance (ANOVA) was carried out when the data had a normal distribution and variance equality, followed by Tukey’s post hoc test. Otherwise, the data were compared using Welch’s tests. Experimental data without normal distributions were analyzed using a non-parametric Kruskal–Wallis ANOVA with Dunn’s test. Data were considered statistically significant when *p* < 0.05. Statistical analysis and illustration were conducted using GraphPad Prism 9 (GraphPad Software Inc., Boston, MA, USA).

## 3. Results

### 3.1. Contents of Main Energy Substances in the Liver of S. hasta under Starvation Stress

**Figure 1:**
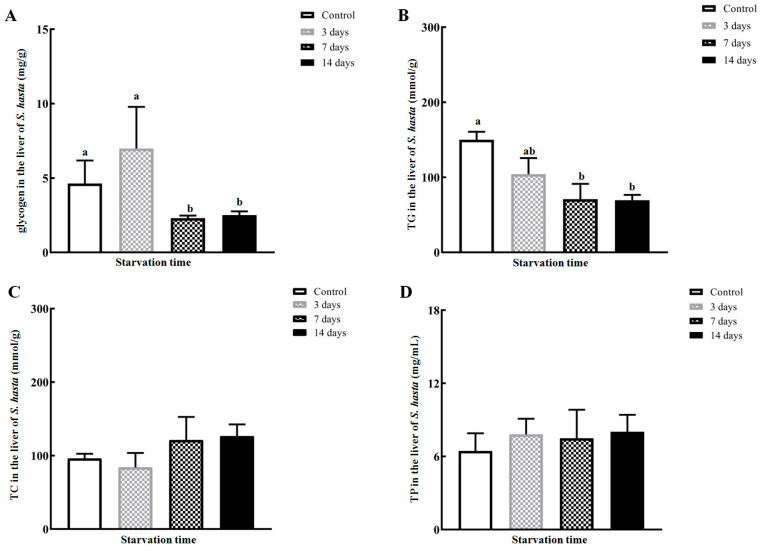
Hepatic biochemical parameters of major energy storage in *Synechogobius hasta* experiencing starvation. The average contents of glycogen (**A**), total triglycerides (**B**), total cholesterol (**C**), and total protein (**D**) in the livers of *S. hasta* which had fasted for different periods. The results are presented as means ± SD (*n* = 5) and were evaluated using a one-way ANOVA. Different superscript letters indicate significant differences (*p* < 0.05). The abbreviations for the parameters in Figure 1 are as follows: TG: triglycerides; TC: total cholesterol; TP: total protein.

During the entire experimental process, no deaths or obvious adverse signs were found in any groups. The mean content of hepatic glycogen was evidently lower as the fasting time extended to 7–14 days (*p* < 0.05; Figure 1A). The hepatic TG level was gradually reduced and reached its minimum on day 14 of fasting (*p* < 0.05; Figure 1B) at only 46.3% of that in the control *S. hasta*. However, food deprivation did not significantly influence the TC or TP concentrations in the *S. hasta* livers (*p >* 0.05; Figure 1C,D).

### 3.2. Intermediate Metabolic Enzyme Activity in the Liver of S. hasta under Starvation Stress

**Figure 2:**
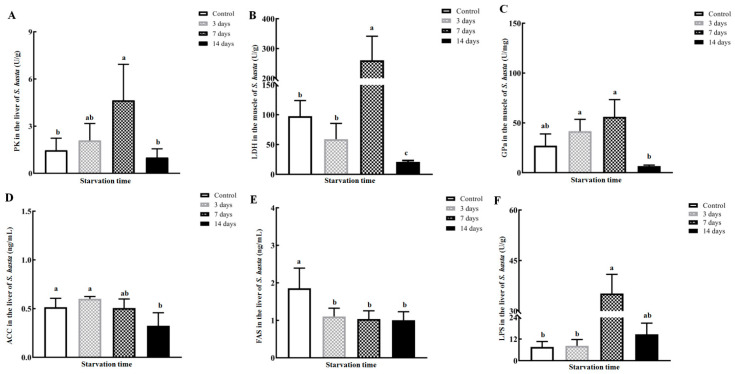
Activities of hepatic enzymes associated with glucose and lipid metabolism in *Synechogobius hasta* experiencing starvation. The average enzymatic activities of PK (**A**), LDH (**B**), GPa (**C**), ACC (**D**), FAS (**E**), and LPS (**F**) in the livers of *S. hasta* fasted for different periods. The results are presented as means ± SD (*n* = 5) and were evaluated using a one-way ANOVA or a Kruskal–Wallis ANOVA. Different superscript letters indicate significant differences (*p* < 0.05). The abbreviations for the parameters in Figure 2 are as follows: PK: pyruvate kinase; LDH: lactate dehydrogenase; GPa: glycogen phosphorylase a; ACC: acetyl-CoA carboxylase; FAS: fatty acid synthase; LPS: lipase.

Hepatic PK and GPa increased stepwise and reached their highest levels on day 7 of fasting (*p* < 0.05). They dropped robustly to the level of the control group on the 14th day of starvation (*p* < 0.05; Figure 2A,C). Similarly, LDH exhibited a peak on the seventh day of fasting, followed by a significant decline to a minimum below the level of the control group (*p* < 0.05; Figure 2B). FAS activity underwent substantial down-regulation in 3-day fasted fish and consistently maintained this lower value during days 3–14 of starvation (*p* < 0.05; Figure 2E). Liver ACC activity was relatively constant over the beginning and middle of fasting but markedly declined on the 14th day of the fasting treatment (*p* < 0.05; Figure 2D). In contrast, hepatic LPS was obviously elevated with an 8.42-fold enhancement in 7-day starved *S. hasta* and maintained this higher activity until the end of the experiment (*p*< 0.05; Figure 2F).

### 3.3. Antioxidant Properties in the Liver of S. hasta under Starvation Stress

**Figure 3:**
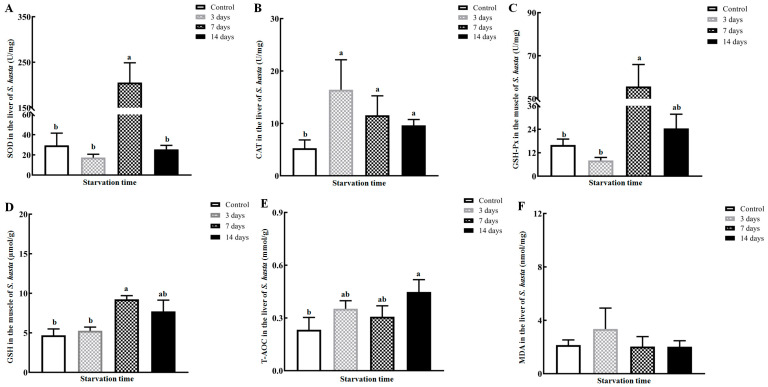
Hepatic antioxidant parameters of *Synechogobius hasta* experiencing starvation. The average levels of SOD (**A**), CAT (**B**), GSH-Px (**C**), GSH (**D**), T-AOC (**E**), and MDA (**F**) in the livers of *S. hasta* fasted for different periods. The results are presented as means ± SD (*n* = 4–5) and were evaluated using a one-way ANOVA or a Kruskal–Wallis ANOVA. Different superscript letters indicate significant differences (*p* < 0.05). The abbreviations for the parameters in Figure 3 are as follows: SOD: superoxide dismutase; CAT: catalase; GSH-Px: glutathione peroxidase; GSH: glutathione; T-AOC: total antioxidative capacity; MDA: malondialdehyde.

SOD activity dramatically increased in the *S. hasta* livers to a maximum (over 205 U/mg) after 7 days of food shortage, then fell back to near the control value in the group starved for 14 days (*p* < 0.05; Figure 3A). CAT was significantly elevated as early as after 3 days of starvation and maintained its higher activity until the end of fasting (*p* < 0.05; Figure 3B). The levels of liver GSH-Px and GSH showed apparent increases and peaks on day 7 of food deprivation (*p* < 0.05; Figure 3C,D), followed by slight reductions in *S. hasta* which had endured 14-day fasting. The hepatic T-AOC progressively increased to its maximal value over days 3 to 14 of starvation (*p* < 0.05; Figure 3E). Only the MDA content was not significantly altered at all time points of the fasting trial (*p >* 0.05; Figure 3F).

### 3.4. Morphological Alterations in the Liver of S. hasta under Starvation Stress

**Figure 4:**
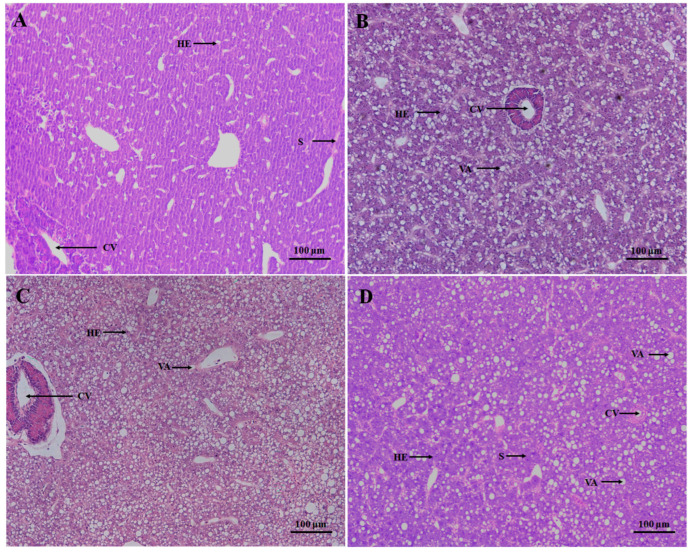
Hepatic morphology of *Synechogobius hasta* experiencing starvation. Representative histological sections of liver tissues from *S. hasta* fed continually (**A**) or exposed to 3 (**B**), 7 (**C**), or 14 (**D**) days of fasting. The liver sections were stained with a hematoxylin and eosin solution. HE: hepatocytes; CV: central vein; VA: vacuolization; S: sinusoids; scale bar: 100 μm.

The hepatocytes in the control *S. hasta* had a tight, orderly arrangement with a regular morphology and no obvious abnormalities (Figure 4A). After 3 days of starvation, the livers exhibited well-demarcated cell boundaries, clearly structured central veins, and radially arranged sinusoids with a few vacuolated structures in some regions (Figure 4B). More cytoplasm vacuolations, larger intercellular spaces, and some congestions and hyperplasia of the epithelial lining around the local central veins appeared in the livers of 7-day starved *S. hasta* (Figure 4C). As the fasting was prolonged to 14 days, the hepatic cytoplasm vacuoles decreased in amount and volume, accompanied by some nucleus migration, nuclear atrophy, and inflammatory infiltration within the central veins (Figure 4D). Simultaneously, PAS-stained *S. hasta* livers exhibited a lighter color of glycogen granules with the extension of fasting (Figure S1).

### 3.5. Relative Expression of Glucose and Lipid Metabolism-Related Genes in the Liver of S. hasta under Starvation Stress

**Figure 5:**
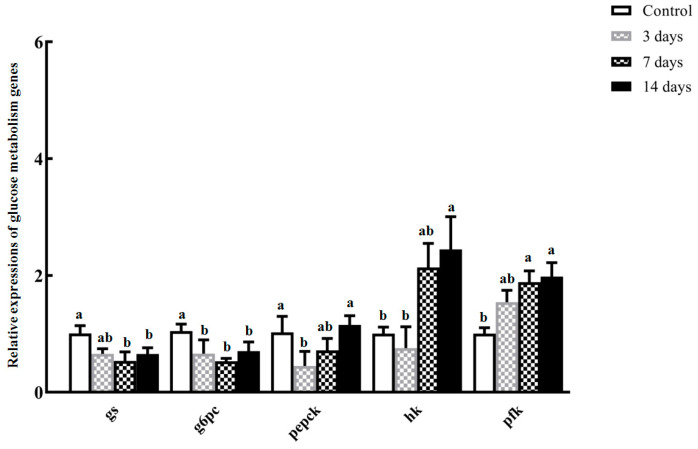
Hepatic expression of glucose metabolism-associated genes in *Synechogobius hasta* experiencing starvation. Relative expression abundances of representative genes involved in glucose metabolism in the livers of *S. hasta* which had fasted for different periods. The transcript level of each gene was determined via qRT-PCR and normalized to the level of an internal reference gene (*β-actin*). The qRT-PCR results are presented as means ± SD (*n* = 5) and were evaluated using a one-way ANOVA. Different superscript letters indicate significant differences (*p* < 0.05).

Upon initiating starvation, both glycogen synthase (*gs*) and glucose-6-phosphatase (*g6pc*) genes were substantially down-regulated (*p* < 0.05; Figure 5). The mRNA of phosphoenolpyruvate carboxykinase (*pepck*) gene rapidly decreased to its lowest value in 3-day fasted *S. hasta* (*p* < 0.05) and was subsequently up-regulated to levels comparable to the control group at 7–14 days post-starvation (Figure 5). Hexokinase (*hk*) and phosphofructokinase (*pfk*) gene expression showed the progressive elevation during the food shortage, with peaks in the livers of 14-day starved *S. hasta* (*p* < 0.05; Figure 5).

**Figure 6:**
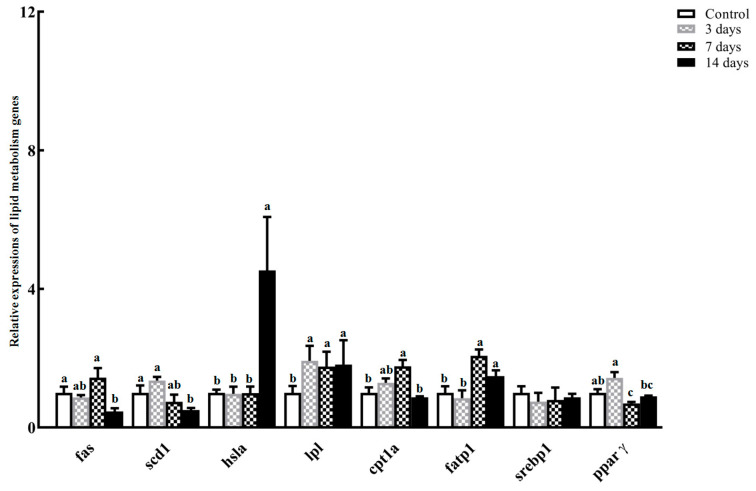
Hepatic expression of lipid metabolism-associated genes in *Synechogobius hasta* experiencing starvation. Relative expression abundances of representative genes involved in lipid metabolism in the livers of *S. hasta* fasted for different periods. The transcript level of each gene was determined via qRT-PCR and normalized to the level of an internal reference gene (*β-actin*). The qRT-PCR results are presented as means ± SD (*n* = 5) and were evaluated using a one-way ANOVA. Different superscript letters indicate significant differences (*p* < 0.05).

Two lipogenic genes, fatty acid synthase (*fas*) and stearoyl-CoA desaturase 1 (*scd1*), exhibited relatively stable abundances during the early and middle phases of starvation (*p* > 0.05), whereas they were dramatically down-regulated to levels lower than those of the other groups at 14 days post-fasting (*p* < 0.05; Figure 6). The hormone-sensitive lipase a (*hsla*) gene was strongly up-regulated on the 14th day of starvation (*p* < 0.05; Figure 6). Conversely, dramatically higher levels of carnitine palmitoyltransferase 1 a (*cpt1a*) mRNA were observed between 3 and 7 days of starvation (*p* < 0.05; Figure 6). In *S. hasta* liver, lipoprotein lipase (*lpl*) and fatty acid transport protein 1 (*fatp1*) genes were significantly elevated to varying degrees as starvation continued, particularly over 3 days of starvation (*p* < 0.05, Figure 6). The relative abundance of peroxisome proliferator-activated receptor γ (*ppar γ*) obviously decreased when starvation reached or exceeded 7 days (*p* < 0.05; Figure 6). Additionally, sterol regulatory element binding protein 1 (*srebp1*) gene was not transcriptionally altered during the fasting period (*p* > 0.05; Figure 6).

## 4. Discussion

### 4.1. Effect of Starvation Stress on the Mobilization of the Main Energy Reserves in the Liver of Synechogobius hasta

Food deficiencies in natural and artificial conditions can provoke the sequential consumption of endogenous fuels and adaptively reorganize the energetic metabolism in the body [1,2,5]. This is the energy strategy by which fish deal with starvation. In this study, hepatic glycogen and TG were dramatically reduced when fasting was extended to 7 days or more (Figure 1), indicating the breakdown of carbohydrates and fats stored in the *S. hasta* livers, starting on the 7th day of starvation. This coincided well with a study on food-deprived gibel carp (*Carassius auratus gibelio* var. CAS III) [23] in which substantial declines in hepatic glycogen and TG were detected after 7 days of starvation. Compared with other lipid metabolites that could be utilized as energy supplies in fish [24,25], TG are a more easily accessible source of fat reserves during food deficiency [26]. Therefore, the amount of TC, another major lipid component, was not altered in *S. hasta* liver during the same period of fasting (*p* > 0.05; Figure 1), and its consumption would likely be seen under long-term hunger. Moreover, liver protein did not change during the food deprivation period (Figure 1), consistent with the results of Siberian sturgeon (*Acipenser baerii*) subjected to 2 weeks of food scarcity [27], as well as muscular protein data in fasted *S. hasta* [17]. The conservation of protein amount in the liver of *S. hasta* suggested physiological tactics to minimize protein losses in fish facing food shortages [1,5]. It might also be attributed to the protein-sparing effect induced by the consumption of carbohydrates and fats in the liver [9].

In this study, liver lipids seemed to be preferentially consumed by the starving *S. hasta* compared with liver glycogen, showing the reduced TG content as early as the 3rd day of fasting. Additionally, the consumption of hepatic TG was slightly higher than that of hepatic glycogen. This is different from the preferential depletion of glycogen as an energy source in the liver of largemouth bass (*Micropterus salmoides*) [9] and rainbow trout (*Oncorhynchus mykiss*) [6] during short-term starvation, as well as in the muscle of *S. hasta* [17] under short-term fasting. Apart from species divergence, the core role of the liver in lipid homeostasis in fish [1,5] and the massive hepatic fat storage in *S. hasta* [20] which could sufficiently cover the energy needs for fasted *S. hasta*, at least over the short term, may account for this observation being inconsistent with previous reports. Therefore, we might speculate that glucose and lipids, particularly TG, serve as the preferential energy-supplying substances in the *S. hasta* liver, a conventional energy strategy during starvation in multiple fish species [1,5,13,24,28,29] if fasting persists for less than 14 days.

### 4.2. Effect of Starvation Stress on the Key Enzyme Activity Related to Glucose and Lipid Metabolism in the Liver of Synechogobius hasta

As hunger extended to 7 days, the activities of glycogen phosphorylase a (GPa) and pyruvate kinase (PK) in the *S. hasta* livers gradually increased to their respective peaks (Figure 2), catalytically generating more glycogen breakdown substrates and pyruvate. These products can be further utilized as fuel or intermediary substrates via the glycogenolysis and glycolysis pathways [30] in response to fasting, particularly at the initial and intermediate phases of fasting. However, substantially reduced hepatic glycogen is foreseeable under long-term hunger [1,5,30]. Coupled with the lower energy density of carbohydrates [15], this inevitably triggers a metabolic shift to other physiological fuels and in turn hinders glycolysis and/or pentose phosphate in fish [1,30]. In this study, the hepatic PK and GPa activities were markedly reduced in the 14-day starved group (Figure 2), which partly contributed to glycemic stability under food restriction [1,30,31,32]. Similar changes were demonstrated in fasted rainbow trout [30] and fasted sturgeon [1,31], particularly when enduring food shortages shorter than 20 days.

A large increase in hepatic lactate dehydrogenase (LDH) was observed after 7 days of starvation (Figure 2), indicating increased lactate production. Given that pyruvate from glycolysis tends to generate lactate as a fast energy supply under acute energy stress [33,34], this increase in LDH in *S. hasta* could partly compensate for lactate depletion during the early phase of fasting and exacerbated liver damage, consistent with a previous report [35] and histological results in *S. hasta*. However, the lactate produced in the liver and other tissues is quickly consumed via the TCA cycle to maintain tight homeostasis of lactate flux. Therefore, a dramatically lower LDH level was observed on the 14th day of fasting (Figure 2). Considering that ATP and relevant intermediate metabolites generated by lactate recycling and turnover are less than that of mitochondrial oxidative phosphorylation [33], more pyruvate may directly enter the TCA pathway for higher energy production during prolonged nutritional deprivation. This speculation could be partly verified by dampening the function of LDH, where more pyruvate flows directly into the TCA cycle for ATP production [36] rather than utilizing lactate turnover as a central carbon donor.

Diminished biosynthesis of energy substrates, including lipids, is an inevitable consequence of food scarcity in fish [1,5,37]. In this study, the repression of hepatic lipogenesis was partly reflected by the lower activities of acetyl-CoA carboxylase (ACC) and fatty acid synthase (FAS), especially in the individuals fasted for 14 days (Figure 2). This agreed with the decreased activity/expression of key lipogenic enzymes in the livers of multiple fish species during food deprivation [1,38,39,40,41]. Conversely, a substantial rise in hepatic lipase (LPS) activity appeared in the mid-to-late phase of fasting (Figure 2), indicating the initiation of hepatic fat decomposition in *S. hasta* starved for 7 days or more. Up-regulation of LPS and LPS superfamily members was also demonstrated in the livers of other fish at the mRNA/protein levels [23,24,38,39,40] when they faced starvation for 7 days or longer.

### 4.3. Effect of Starvation Stress on the Antioxidant Capacity in the Liver of Synechogobius hasta

It has been verified that the antioxidant defense system in fish tissues is influenced by nutritional status, including starvation [1,3,5,6]. In our work, the hepatic activities of SOD and CAT, typical antioxidant enzymes, were markedly enhanced in the food-deprived groups to varying degrees, particularly after 7 days of fasting (Figure 3). These up-regulated enzymes could scavenge more superoxide anions (O2·-), hydroxyl radicals (·OH), and hydrogen peroxide (H_2_O_2_) in direct or indirect ways [5] and in turn ameliorate hunger-induced oxidative damage/reactive oxygen species (ROS) within the *S. hasta* liver [17]. Their increasing trends were aligned with prior studies in other marine and freshwater species subjected to nutritional privation less than 2 weeks [24,42,43,44,45,46]. Contrarily, the SOD and CAT activities were decreased in the liver of Adriatic sturgeon (*Acipenser naccarii*) and rainbow trout experiencing 2–10 days of hunger [47]. Besides inter-species variations, organ-specific sensitivity to starvation may elicit the above-mentioned inconsistency. This speculation was somewhat corroborated by the finding that CAT was not altered in the extrahepatic tissues of *S. hasta* and other fish during fasting [17,48].

GSH-Px and its reaction substrate GSH were considerably increased in the livers of *S. hasta* fasted for 7–14 days (Figure 3), which could mitigate ROS-triggered damage following food deprivation in a synergistic manner [5]. Moreover, these two components cooperatively contribute to the removal of lipid peroxidation products within cells [49], preventing excessive lipid peroxidation, lipotoxicity, and other metabolism disorders during food scarcity. However, their elevation strongly depends on the availability of glutathione and related molecules. A depleted endogenous glutathione pool under persistent starvation well beyond 15 days may fail to effectively neutralize ROS via the antioxidant system. This induces the intensified lipid peroxidation [6] and a vicious spiral of oxidative damage [46], inevitably causing a lowering trend in hepatic GSH-Px and GSH in *S. hasta* during the late stage of fasting. GSH-Px and GSH were observed to increase first and then decrease in other starving fishes [1,3,17,24,50], which could support our results.

No change in hepatic MDA was found with increasing durations of food deprivation (Figure 3), similar to the results observed when African catfish (*Clarias gariepinus*) endured 2 weeks of fasting [50]. It might be interpreted with a protective role of antioxidant system due to the relatively high levels of hepatic CAT, GSH-Px, and GSH observed at the late phase of fasting. High levels of enzymatic and non-enzymatic antioxidants in the *S. hasta* livers are resilient enough to alleviate oxidative stress and induced lipid peroxidation when challenged by a short-term nutrient shortage (below 14 days). In *S. hasta* livers, the gradually elevating T-AOC, a parameter indicating the overall antioxidant ability, could also verify this view to some degree. Certainly, the overall antioxidative functioning of a fish’s body may ultimately weaken due to antioxidant storage exhaustion and oxidative stress provoked by continuous energy mobilization during prolonged food restriction [51], leading to liver stress and damage, as confirmed in many fish suffering severe hunger [1,5].

### 4.4. Effect of Starvation Stress on the Hepatic Morphology in Synechogobius hasta

In parallel with the physio-biochemical and metabolic adjustments to energetic scarcity, histological alterations were evident in the *S. hasta* livers from day 3 of starvation and became more visible with extended starvation to day 7 (Figure 4). The livers exhibited less vacuolization and smaller size of hepatocytes, but showed more nucleoli atrophy, local dissolution of some hepatocytes, and other structural irregularities on the 14th day (Figure 4). These results were similar to the hepatic histological modifications observed in several fish species experiencing short-term food deprivation [28,52,53,54,55]. Hunger-induced microstructural abnormalities in visceral organs also emerged in the gut and muscle of *S. hasta* starved for 3–14 days [8,17]. Furthermore, PAS-positive hepatic glycogen tended to be lightened/reduced in *S. hasta* exposed to 7–14 days of dietary scarcity (Figure S1). The morphological responses to nutrient shortages in the *S. hasta* liver might be linked to the consumption and reallocation of hepatic endogenous carbohydrates and fat. This speculation could be partly validated by changes in the contents of energy reserves and gene expression related to glucose and lipid metabolism in this study. Noticeably, long-term and excessive starvation could cause structurally irreversible damage to the liver, which would not be rapidly restored by re-feeding [5,52], consequently impairing normal hepatic function and even survival in *S. hasta*.

### 4.5. Effect of Starvation Stress on the Transcriptional Expression of Glucose and Lipid Metabolism-Associated Genes in the Liver of Synechogobius hasta

A blocked supply of exogenous food impedes the anabolic process and accumulation of the corresponding nutrients in fish [1,5]. As anticipated, hepatic transcripts of glycogen synthase (GS) were reduced significantly post-fasting (Figure 5), directly suppressing the glycogen biosynthetic reaction. Similar down-regulation was observed for glucose-6-phosphatase (G6PC) (Figure 5), which has an essential role in endogenous glucose production and gluconeogenesis in the liver [56,57]. Thus, less endogenous glucose could be generated in the *S. hasta* liver and/or released into the liver and extrahepatic tissues following hunger. Conversely, phosphofructokinase (PFK) and hexokinase (HK), two regulatory enzymes that catalyze the central step of glycolysis, were transcriptionally increased during starvation and peaked in the 14-day fasted individuals (Figure 5), implying an enhanced glycolytic process in the *S. hasta* liver under starvation. This partly agreed with the food deprivation-induced up-regulation of *pfk* and *hk* expression/activity in the livers of Japanese flounder [15], largemouth bass (*Micropterus salmoides*) [9], and large yellow croaker [58], particularly during brief/short-term starvation.

The gluconeogenic pathway is dually regulated by two core enzymes (phosphoenolpyruvate carboxykinase (PEPCK) and G6PC) and the glucogenic substrate content in the liver [57], where the former has a more dominant modulatory effect. Under food restriction, lower availability of gluconeogenic precursors within the *S. hasta* liver can be overcome to some extent by intrahepatic non-glucose substances, including endogenous glycerol and glutamine [59,60]. Thus, the down-regulated hepatic *pepck* returned to the control range in the later stage of food deprivation (Figure 5), similar to the *pepck* expression trend in other aquatic animals that endured 2 weeks of fasting [61]. Moreover, given the partially glycolysis-facilitating role of PEPCK that was validated in vitro [60], the recovery of *pepck* expression may contribute to glycolysis in the *S. hasta* liver during the later stage of fasting.

A paucity of food directly diminished the substrates and precursors of fat biosynthesis in the liver, resulting in significantly lower expression of *fas* and stearoyl-CoA desaturase 1 (*scd1*) genes, especially during later fasting stress (beyond 7 days). The expression patterns of two critical lipogenic genes were consistent with the reduced hepatic expressions/activities of *fas* and *scd1* in multiple aquatic animals facing nutrient shortages [24,38,39,40,62]. The reduced levels of liver TG and ACC detected in this fasting trial partially confirmed these expression results. Additionally, the *S. hasta* liver exhibited a pattern of *scd1* expression that was basically consistent with that observed in food-deprived *S. hasta* muscle and intestine, while *fas* transcription was discordant [8,17], providing further evidence of the tissue-dependent responses of lipogenic genes in fish during food deprivation [38,40,48].

The hepatic mRNA expression of lipoprotein lipase (LPL), which decomposes chylomicron-derived TG and functions as a ligand/bridging factor for intracellular uptake of lipid components [38,40], was notably up-regulated after 3–14 days of starvation (Figure 6). Similarly, hormone-sensitive lipase (HSL), as an essential rate-limiting enzyme for lipolysis, showed strong elevation in mRNA in *S. hasta* starved for 14 days (Figure 6). More lipid-related substrates would be generated by boosting *hsl-* and *lpl*-mediated fat breakdown in the *S. hasta* liver during prolonged food deficiency. Among them, fatty acids can be transported to appropriate intracellular locations within the livers of fasted *S. hasta* to increase energy substrates by significantly up-regulating fatty acid transport protein 1 (*fatp1*) (Figure 6) and fatty acid binding protein 1 (*fabp1*) (Figure S2). Transcriptional/post-transcriptional increases in Fatp and Fabp family members that mediate trans-membrane fatty acid transportation and uptake during ongoing starvation were also documented in the livers of many marine and freshwater fish [18,58,63,64].

Unlike unaltered sterol regulatory element binding protein 1 (*srebp1*) gene, mRNA level of peroxisome proliferator-activated receptor γ (PPAR *γ*), another well-known transcription factor governing lipid metabolism signaling [8,65], was obviously diminished in the livers of *S. hasta* experiencing non-feeding for 7–14 days (Figure 6). Their expression trends, along with the same patterns as *srebp1* and *ppar γ* in the intestines and muscles of starved *S. hasta* [8,17], further corroborated that PPAR γ signaling is more dominant in mediating lipid homeostasis than the SREBP1 pathway in the metabolically active tissues of fish [8,11,66]. Given the comprehensive changes in lipid metabolism-associated genes in this study, it can be inferred that fasting treatment both enhanced lipolytic and transport activities and attenuated lipid synthesis in the liver via the PPAR γ-driven lipid metabolism pathway, thereby causing lower fat storage in the *S. hasta* liver. This was supported by a reduction in hepatic TG in *S. hasta*, which also echoed previous findings that hunger activates lipolysis and oxidation, suppresses lipogenesis, and is closely linked with a decline in TG in food-deprived fish [9,23,24,62].

## 5. Conclusions

Hepatic glycogen and TG both markedly declined in *S. hasta* upon starvation, and this was accompanied by an increase in liver antioxidant performance to some extent and hepatic morphological alterations. With prolonged fasting, the PK, LDH, and GPa activities first increased and then decreased due to the rather limited energy generated solely via hepatic glycogen decomposition. Lipogenic enzymes were considerably reduced, while LPS was increased in the liver of *S. hasta* post-starvation. Furthermore, food scarcity transcriptionally suppressed glucose and lipid anabolism and enhanced genes related to the catabolic pathway and transport in *S. hasta* liver. Collectively, these results indicated that *S. hasta* subjected to food deprivation could simultaneously utilize hepatic carbohydrates and lipids by accelerating the decomposition and trafficking of glucose and lipids as well as dampening their respective biosynthesis pathways, thereby contributing to energetic homeostasis and the function of the liver during prolonged starvation.

## Figures and Tables

**Table 1:** Specific primers for qRT-PCR assay.

Gene	Forward Primer (5′-3′)	Reverse Primer (5′-3′)
*g6pc*	ATTTAGCCTCGCCTCGTCAG	CCAATCACAGCCACCCAGAT
*gs*	CTGACCCCATCCTGACCAAC	CAGCCACGCACAAAGTCATC
*h* *k*	ACATGGAGGAGCTGCGTAAC	TGTCCAAGGCTCCATCATCT
*pepck*	TGTGGATATGGGTGCGCTTT	TCCAACTGCCTCAACTCGTC
*pfk*	TCTCCAAGAAACTCACCCGC	CCTGCGTATCTTCATGGCCT
*fas*	CATCATCACTGGAGGTCTTGGA	TACGAATGCCTGATCTGGAAGT
*scd1*	GACAACCAGCCCAAATCC	GAGCCCCATCAGAAAGAC
*hsla*	CTATGGTGAGACCTACGGTAAAC	TCCTGCTAAAGCCTGTGATT
*lpl*	AGTCCGATCAACACGAAGC	GGTGCCGTTCCCATTTAG
*cpt1a*	CGCTCCTGCTCCAATGAGA	GAGACCACATAGAGGCAGAAGA
*fatp1*	CCACTGGGCTCAGAATCAAG	CAAGTTCAGCTCCAAAGACAATA
*srebp1*	TGCTATGCGGAGGTTATTCATC	GTTGCTCTGCGTCGTAGTG
*ppar γ*	TTCTTCCACAGTTGCCAGTC	GTTCATCAGAGGAGCCATCA
*18s*	TTCGATGGTACTTTCTGTGC	CTGCCTTCCTTGGATGTG
*α-tubulin*	CACTTCCCTCTTGCCACCTA	ACGGTACAGGAGACAACAGG
*β-actin*	GTGCGTGACATCAAGGAGAAG	CGAGGAAGGATGGCTGGAA

Note: The sequences of specific primers in this study were designed using the Primer Premier 5 or described in previous research [8,17,18,21]. The abbreviations in Table 1 were as follows: *g6pc*: glucose-6-phosphatase; *gs*: glycogen synthase; *hk*: hexokinase; *pepck*: phosphoenolpyruvate carboxykinase; *pfk*: phosphofructokinase; *fas:* fatty acid synthase; *scd1*: stearoyl-CoA desaturase 1; *hsla*: hormone-sensitive lipase a; *lpl*: lipoprotein lipase; *cpt1a*: carnitine palmitoyltransferase 1 a; *fatp1*: fatty acid transport protein 1; *srebp1*: sterol regulatory element binding protein 1; *ppar γ*: peroxisome proliferator-activated receptor γ; *α*-*tubulin*: alpha-tubulin; *β-actin*: beta-actin.

## Data Availability

All data used for this study are presented in the main paper and the Supplementary File online.

## Supplementary Materials

The following supporting information can be downloaded at: https://www.mdpi.com/article/10.3390/ani14182734/s1, Figure S1: histological structure of PAS-stained hepatic section from *Synechogobius hasta* experiencing starvation; Figure S2: hepatic expression of fatty acid binding protein 1 (*fabp1*) gene in *Synechogobius hasta* experiencing starvation; Material S1: procedures of periodic acid-schiff (PAS) staining.
