# Supplementary material for: Influence of Starvation on Biochemical, Physiological, Morphological, and Transcriptional Responses Associated with Glucose and Lipid Metabolism in the Liver of Javelin Goby (Synechogobius hasta)
Source: Animals (Basel). 2024 Sep 21;14(18):2734. doi: 10.3390/ani14182734 (PMC11429288; doi:10.3390/ani14182734)
Supplement: Supplementary file 1 [file animals-14-02734-s001.zip › Figure. S1 for Animals.pdf]

Figure S1.

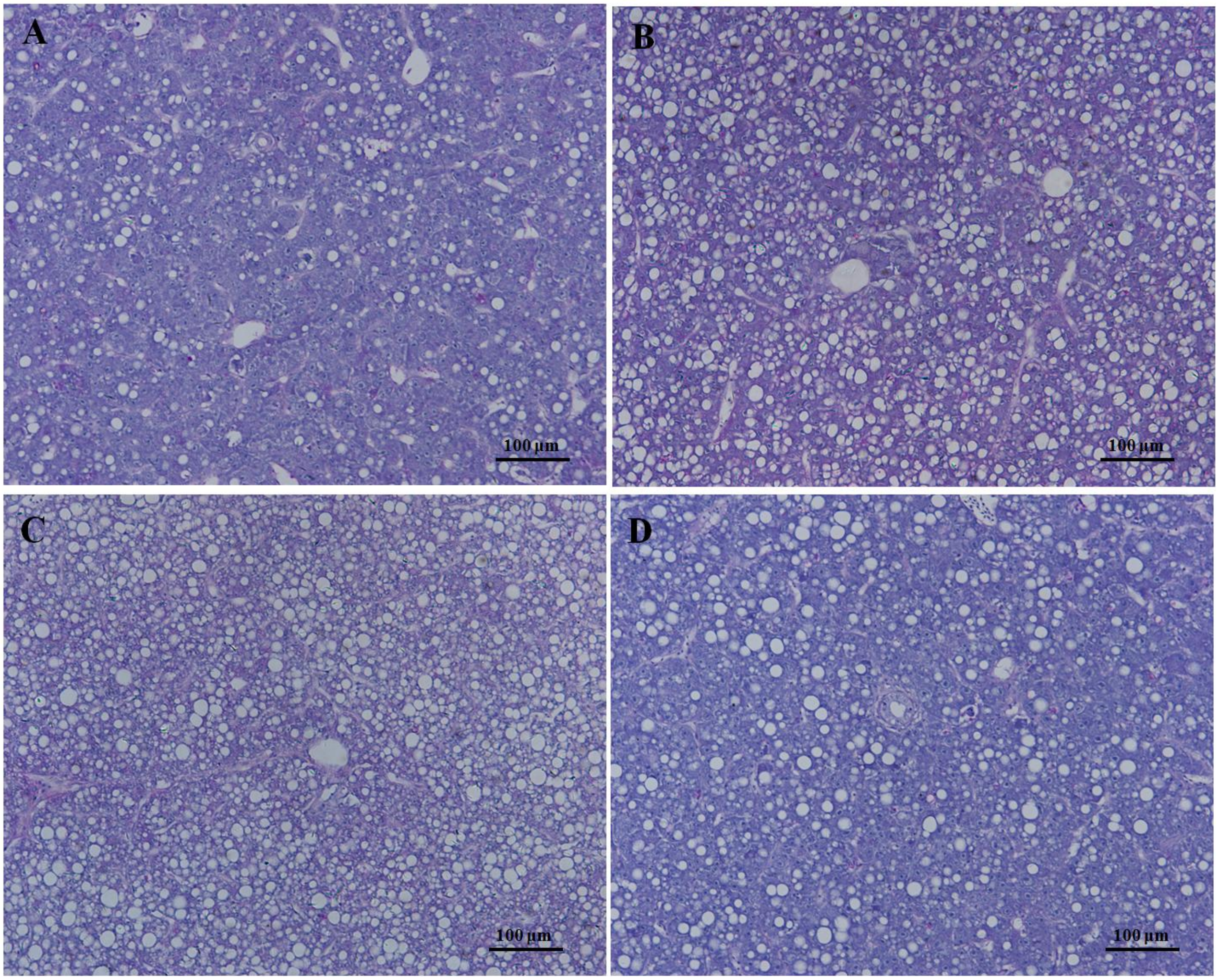

Figure S1. Histological structure of PAS-stained hepatic section in *Synechogobius hasta* experiencing starvation.

Representative photographs of liver sections in *S. hasta* experiencing 0 (A), 3 (B), 7 (C), and 14 (D) days of starvation. Hepatic sections were stained with periodic acid-Schiff.

Scale bar: 100 µm.
