# Supplementary material for: Influence of Starvation on Biochemical, Physiological, Morphological, and Transcriptional Responses Associated with Glucose and Lipid Metabolism in the Liver of Javelin Goby (Synechogobius hasta)
Source: Animals (Basel). 2024 Sep 21;14(18):2734. doi: 10.3390/ani14182734 (PMC11429288; doi:10.3390/ani14182734)
Supplement: Supplementary file 1 [file animals-14-02734-s001.zip › Figure. S2.pdf]

Figure S2.

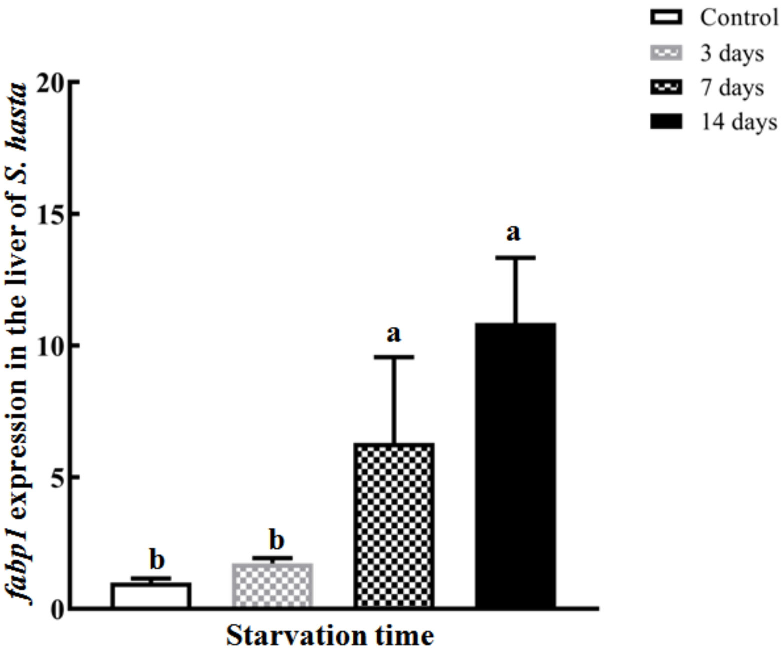

Figure S2. Hepatic expression of fatty acid binding protein 1 (*fabp1*) genes in *Synechogobius hasta* experiencing starvation.

Relative expression abundances of *fabp1* in the livers of S. hasta fasted for different periods. The transcript level of each gene was determined via qRT-PCR and normalized to the level of an internal reference gene (*β-actin*). The qRT-PCR results are presented as means ±SD (n = 5) and were evaluated using a one-way ANOVA. Different superscript letters indicate significant differences ($P < 0.05$).
