# Supplementary material for: Influence of Starvation on Biochemical, Physiological, Morphological, and Transcriptional Responses Associated with Glucose and Lipid Metabolism in the Liver of Javelin Goby (Synechogobius hasta)
Source: Animals (Basel). 2024 Sep 21;14(18):2734. doi: 10.3390/ani14182734 (PMC11429288; doi:10.3390/ani14182734)
Supplement: Supplementary file 1 [file animals-14-02734-s001.zip › Material S1 for Animals.pdf]

Procedures of periodic acid-schiff (PAS) staining

The brief steps of PAS staing are as follows.

Paraffin sections were dewaxed in xylene solution and then rehydrated with a gradient alcohol to distilled water. The hepatic sections were covered with the fresh periodic acid solution and incubated at RT for 5 min, followed by a reaction with Schiff's reagent for 20 min. After rinsing thoroughly in running water, hepatic slides were counterstained with the hematoxylin solution at RT for 3-5 min. Finally, the slices were dehydrated, air-dried slightly, and sealed with neutral gum.

PAS-stained hepatic sections were observed and photographed under a Nikon 90i Microscope (Nikon, Tokyo, Japan).
